# Disruption of Pituitary Gonadotrope Activity in Male Rats After Short- or Long-Term High-Fat Diets Is Not Associated With Pituitary Inflammation

**DOI:** 10.3389/fendo.2022.877999

**Published:** 2022-04-13

**Authors:** Ghislaine Garrel, Claude Rouch, David L’Hôte, Salma Tazi, Nadim Kassis, Frank Giton, Julien Dairou, Pascal Dournaud, Pierre Gressens, Christophe Magnan, Céline Cruciani-Guglielmacci, Joëlle Cohen-Tannoudji

**Affiliations:** ^1^Université Paris Cité, CNRS, Inserm, Unité de Biologie Fonctionnelle et Adaptative, Paris, France; ^2^Université Paris Cité, CNRS, Unité de Biologie Fonctionnelle et Adaptative, Paris, France; ^3^AP-HP, Pôle biologie-Pathologie Henri Mondor, Inserm IMRB U955, Créteil, France; ^4^Université Paris Cité, CNRS, Laboratoire de Chimie et de Biochimie Pharmacologiques et Toxicologiques, Paris, France; ^5^Université Paris Cité, Neurodiderot, Inserm, Paris, France

**Keywords:** pituitary, inflammation, high-fat diet, gonadotropin, fatty acids, omega 3

## Abstract

Overnutrition is associated with the activation of inflammatory pathways in metabolically linked organs and an early hypothalamic inflammation is now known to disrupt the central control of metabolic function. Because we demonstrated that fatty acids (FA) target the pituitary and affect gonadotropin synthesis, we asked whether overnutrition induces pituitary inflammation that may contribute to obesity-associated disorders in the control of reproduction. We analyzed pituitary inflammation and hypothalamic-pituitary-testicular axis in male rats fed a short- (4 weeks) or long-term (20 weeks) high-fat diet. The effect of diet enrichment with the ω3 polyunsaturated FA, DHA, was also analyzed. After only 4 weeks and before weight gain of rats, high-fat diet caused a significant decrease in pituitary gonadotropin and hypothalamic GnRH transcript levels despite unchanged testosterone and inhibin B levels. Contrasting with the hypothalamus, there was no concomitant increases in gene expression of pituitary inflammatory mediators and even a reduction of prototypical cytokines such as interleukin-1β and TNF-α. No inflammation was still detected in the pituitary after 20 weeks although gonadotropin transcripts and circulating levels were still altered. Gonadotropins were the only pituitary hormones remaining affected at this stage of the regimen, underlying a differential susceptibility of pituitary lineages to metabolic disorders. DHA enrichment of the diet did not prevent alterations of gonadotrope activity due to either a long- or a short-term high-fat diet although it blocked early hypothalamic inflammation and attenuated several metabolic effects. Taken together, our findings suggest that high-fat diet-induced defects in gonadotrope activity in male rats occurred despite a lack of pituitary inflammation.

## Introduction

The success of reproduction in mammals is strictly dependent on the synthesis and release of several hormones emanating from the hypothalamic-pituitary-gonadal (HPG) axis and on the endocrine interplay within this axis. Two of the most critical hormones, luteinizing hormone (LH) and follicle-stimulating hormone (FSH), are produced by gonadotrope cells of the anterior pituitary and regulate, in a coordinated fashion, gonadal steroidogenesis and gametogenesis (1). These hormones are glycoproteic heterodimers, composed of a common α-glycoprotein subunit and a specific and rate-limiting β-subunit that confers biological activity. The levels of transcripts coding the three gonadotropin subunits (*Cga*, *Lhb* and *Fshb*) as well as gonadotropin release are tightly regulated by the hypothalamic gonadotropin-releasing hormone (GnRH) (2). In addition to GnRH, other blood carried signals such as steroid hormones and inhibin produced by gonads regulate gonadotropin expression *via* feedbacks to the anterior pituitary. Several metabolically linked factors such as insulin, adipokines, enterokines and nutrients including circulating glucose and fatty acids (FA) have also been shown to target the pituitary in addition to the hypothalamus, and to alter gonadotropin secretion (3–7).

The reproductive activity is closely linked to the metabolic status with both extremes of body weight altering the function of the HPG axis. Diet-induced obesity, whose prevalence has risen dramatically these recent decades, is associated with excess adipose tissue and increased circulating levels of triglycerides and FA (8). Increasing prevalence of obesity has been associated with a higher amount of consumed energy-dense foods and also to changes in the type of consumed fats. Indeed, intake of saturated FA as well as the ratio of ω6/ω3 polyunsaturated FA (PUFA) have significantly increased these last decades (9). Adverse reproductive outcomes have been described in a number of obese patients and include hypo-androgenism and alteration in sperm number and quality in men as well as menstrual dysfunction and pregnancy complications in women (10, 11). Men and women with obesity are more likely to have reduced levels of LH and FSH (10), suggesting that alterations take place at the level of the hypothalamic-pituitary complex. Studies in animal models of diet-induced obesity are consistent with clinical observations in humans. Indeed, a decrease in circulating gonadotropin levels has been reported in several studies conducted on obese rats and mice (12–15).

It is now well-accepted that obesity is associated with a form of chronic, low-grade, sterile inflammation in several metabolically linked peripheral tissues (16, 17). More recently, several studies on animal models of diet-induced obesity have also reported the development of inflammation in several brain areas and notably in the mediobasal hypothalamus, which is crucial for the control of energy homeostasis (18, 19). Hypothalamic inflammation is characterized by increased expression of pro-inflammatory cytokines, macrophage infiltration or glia activation. The hypothalamic inflammatory response appears to develop more rapidly than in the peripheral tissues (18, 20–23) and contributes to weight gain and hypothalamic resistance to leptin and insulin (18, 24). Consistent with these findings in rodents, increased gliosis has been detected by magnetic resonance imaging in the medial hypothalamus of obese patients (21). The crucial role of hypothalamic inflammation in mediating metabolic disorders was further illustrated by experiments showing that inhibition of the inflammatory mediator IKKβ in the mediobasal hypothalamus or loss-of-function mutation of the toll-like receptor (TLR) 4, mediating FA activation of cytokine secretion, protects mice from diet-induced obesity (20, 25). Long-chain saturated FA appear to play a central role in hypothalamic metabolic inflammation. Indeed, intracerebrovascular administration of stearic or palmitic acid was shown to activate TLR2- and TLR4-dependent signaling pathways and to enhance the expression of several pro-inflammatory cytokines within the hypothalamus. In contrast, monounsaturated FA like oleic acid and PUFA, and notably ω3 PUFA, do not do so (20, 26). Moreover, the enrichment of high-fat diets with ω3 PUFA has been shown to partially revert hypothalamic inflammation and body adiposity (27–29).

Although the existence of a diet-induced hypothalamic inflammation has been established, no study so far has investigated whether high-fat diet would also lead to pituitary inflammation. That an inflammation could also take place in the pituitary is however supported by the existence of a lipid sensing in pituitary gonadotrope cells (7). Indeed, FA can activate several signaling pathways including TLR-4 in gonadotrope cells, leading to a modulation of gonadotropin expression and release (14, 30). Furthermore, anterior pituitary cells, and notably the glial-like folliculo-stellate cells, secrete pro-inflammatory cytokines in response to liposaccharide stimulation (31, 32) and the treatment of cultured rat anterior pituitary cells with pro-inflammatory cytokines such as interleukin-1 beta (IL-1β) or tumor necrosis factor-α (TNF-α) was shown to alter basal or stimulated gonadotropin secretion (33–35). Local inflammation of the pituitary may thus contribute to obesity-associated disorders in pituitary endocrine function. The aim of the present study was to investigate in male rats whether a short- (4 weeks) or long -term (20 weeks) high-fat diet leads to inflammation within the pituitary that could be linked to alterations in pituitary gonadotrope activity. Moreover, we also analyzed the effect of an enrichment of the diet with the ω3 PUFA docosahexaenoic acid (DHA) on pituitary inflammation and reproductive parameters.

## Materials and Methods

### Animals and Diets

All procedures were carried out in accordance with the ethical standards of French and European regulations (European Communities Council Directive, 86/609/EEC). Animal use and procedures were approved by the Ethics committee of the Université de Paris and by the French Ministry of Research (approval # 4187-2016021715365460). Four-week-old male Wistar rats were obtained from Janvier Labs (Le Genest-Saint-Isle, France) and housed in pairs in a ventilated cage with water and food *ad libitum* under constant conditions of light (12 h light starting from 7 am). After one week, rats were randomly assigned to control or high-fat diets for 4 (8 rats/group) or 20 (12 rats/group) weeks. Two different isocaloric high-fat diets were used: a high-fat (HF) diet (Safe 246HF, with milk fat as primary fat source) or the Safe 246HF diet supplemented with 5% of the ω3 PUFA, DHA (HF-DHA). Both HF diets contained 45% of total kcal from fat and 37% kcal from carbohydrates and the control diet (Safe A04) only contained 8% of total kcal from fat. The HF diet displayed a ratio of ω6/ω3 PUFA of 5.7/1 whereas the HF-DHA had an ω6/ω3 ratio of 1/1. Source of DHA was marine algae (DHA Gold, Novus International) and DHA accounted for 2.3% of total energy (or 5% of total fat). The detailed composition of the two high-fat diets is indicated in **Supplemental Table 1**. To prevent oxidation, diets were changed every two days. The effects of a high-fat diet enriched with 15% DHA was also measured on some parameters.

At the end of short- or long-term diets, rats were sacrificed between 10 and 12 am by decapitation after isoflurane inhalation. The anterior pituitary glands and mediobasal hypothalamus were rapidly dissected (the mediobasal hypothalamus was delimited laterally by the hypothalamic fissures, anteriorly by a cut anterior to the optic chiasm, and caudally by the infundibular stem). Testis, liver and sub-cutaneous adipose tissue were also dissected from animals that had been on diets for 20 weeks. Tissues were snap-frozen in liquid nitrogen. The blood was allowed to clot for 15 min at room temperature, and then spun down at 1000 g for 20 min to collect the serum. Sera were then stored at -20°C until assays of pituitary and steroid hormones and cytokines levels.

### Metabolic Parameters

Body weight was measured weekly and food intake determined every two days. Body composition of rats (fat and lean masses) was monitored with an Echo Medical Systems EchoMRI 100 (Whole Body Composition Analyzers, EchoMRI, Houston, USA; Functional-Physiological-Exploration platform, BFA unit, Université de Paris) after 12 weeks of diets. The levels of fasting insulin, leptin, glucose and lipids (triglycerides and cholesterol) were determined after 11 weeks of diet using kits following the manufacturer’s specifications. Insulin was assayed with Rat Ultrasensitive Insulin ELISA (ALPCO, Eurobio) and leptin with Mouse/Rat Leptin ELISA kit (BioVendor, Eurobio). Plasma and liver content of triglycerides were determined with the Triglyceride Determination kit (Sigma, TR0100) and cholesterol with the Cholesterol/Cholesteryl Ester Assay kit quantification (Abcam). Circulating glucose was measured from tail blood using a glucometer (Glucofix Tech, A. Menarini diagnostics). The homeostatic model assessment for insulin resistance (HOMA-IR) was calculated as (G_0_)x(I_0_)/22.5 where G_0_ is the fasting glucose level (mmol/L) and I_0_ is the fasting insulin (pmol/L) (36). The Triglyceride-glucose (TyG) index, another index of insulin resistance, was calculated as fasting triglycerides (mg/dL) × fasting plasma glucose (mg/dL)/2 (37). After 13 weeks of diet, rats (8 rats per group) were placed in calorimetric cages (Labmaster, TSE Systems GmbH, Bad Homburg, Germany) to monitor several metabolic parameters such as food intake, spontaneous locomotor activity, energy expenditure (EE), O_2_ consumption and CO_2_ production, respiratory exchange ratio (VCO_2_/VO_2_; RER) (Functional-Physiological-Exploration platform of BFA unit, Université de Paris).

### Real-Time PCR

Total RNAs from rat anterior pituitary, testis, hypothalamus and sub-cutaneous adipose tissue were isolated with the RNeasy-kit (Qiagen, France). Reverse transcription was performed using 1-2 μg RNA in a total volume of 20 μl with Superscript II (Invitrogen) and random primers. Real-time PCRs using Takyon No ROX SYBR master mix (Eurogentec) were carried out in the LightCycler 480 Instrument (Roche Diagnostics) using 5 μl of a mix containing 0.5 μM of each primer and 5 μl of cDNA dilution (1:10 to 1:40). Cycling conditions included an initial heat-denaturing step at 95°C for 3 min, followed by 40 cycles at 95°C for 10 s, 60°C for 10 s and 72°C for 10 s. Oligonucleotide primer sequences are indicated in **Table 1**. Primer pairs were designed to target cDNA fragments encompassing at least one intron in the gene sequence to prevent amplification of genomic DNA. The specificity of amplification was checked by gel electrophoresis and melting curve analysis. Expression levels of genes of interest were determined using the advanced-E-method with standard-curve derived efficiencies obtained from LightCycler 480 software and *Cyclophilin* for normalization.

**Table 1:** Sequences of the primers and probes used for quantification of transcript levels in rat tissues.

Target cDNA	Forward primer written 5’ to 3’ sens	Reverse primer written 5’ to 3’ antisens
***Lhb***	ATCACCTTCACCACCAGCAT	GACCCCCACAGTCAGAGCTA
***Fshb***	TTGCATCCTACTCTGGTGCT	AGCTGGGTCCTTATACAC CA
***Cga***	GCTGTCATTCTGGTCATGCT	GAAGCAACAGCCCATACACT
***Gnrhr***	TCAGCTGCCTGTTCATCATC	AACATTTCCGGATCAAACCA
***Gnrh***	CCAGCACTGGTCCTATGGGT	AGAGCTCCTCGCAGATCCCT
***Kiss1***	CTGCTGCTTCTCCTCTGTGT	AGGCTTGCTCTCTGCATACC
***Il1b***	GCTGTGGCAGCTACCTATGTCTTG	AGGTCGTCATCATCCCACGAG
***Tnfa***	AGATGTGGAACTGGCAGAGG	CCCATTTGGGAACTTCTCCT
***Il6***	TGTTCTCAGGGAGATCTTGG	TCCAGGTAGAAACGGAACTC
***Ccl2***	AGCATCCACGTGCTGTCTC	GATCATCTTGCCAGTGAATGAG
***Ccl5***	ACCACTCCCTGCTGCTTTG	ACACTTGGCGGTTCCTTCG
***Cd68***	ACGGACAGCTTACCTTTGGA	AATGTCCACTGTGCTGCTTG
***Adgre1***	GGACTTCTCCAAGCCTATCGT	CCTCTCAGACTTCTGCTTTGG
***ADAM8***	CAGGCATCGTCATCTACCG	GTAGGCTTGGGTGCCACA
***Ikkb***	TTGTGGGGACCCTGCAATAC	GCCGAAGCTCCAGTAGTCAA
***Nos2***	AAGCACATTTGGCAATGGAGAC	TGGAGCCCAGGCCAAATAC
***Inha***	ATGCACAGGACCTCTGAACC	GGATGGCCGGAATACATAAG
***Inhba***	CAAGGCGGCGCTTCTCAAC	TGCCTTCCTTGGAAATCTCA
***Inhbb***	GAGATCCCGCACCTCGAC	GGTTGCCTTCGTTAGAGACG
***Fst***	CAAGGTTGGCAGAGGTCGCT	CCGAGATGGAGTTGCAAGAT
***Cyclophilin***	CAAAGTTCCAAAGACAGCAG	CTGGCACATGAATCCTGGAA

Cyclophilin was used for normalization.

### Pituitary Hormones and Sex Steroid Assays

Serum LH and FSH concentrations were simultaneously assayed with the Luminex technology using the rat pituitary magnetic bead panel Milliplex Map kit (RPTMAG-86K-Milliplex, Merck-Millipore, Nottingham, UK) following the manufacturer’s instructions. Serum levels of ACTH, PRL and TSH were simultaneously determined using the same technology. Serum levels of GH and Inhibin B were assayed using commercially ELISA kits (Beckman Coulter and Invitrogen, respectively) following manufacturer’s protocols. Serum testosterone and estradiol concentrations were assayed using gas chromatography (GC) coupled with mass spectrometry (GC-MS) procedure, as described previously (38). The linearity of steroid measurement was confirmed by plotting the ratio of the respective steroid peak response/internal standard peak response to the concentration used for calibration standard. Lower limit of quantification was 0.2 pg for estradiol, and 3.9 pg for testosterone.

### Cytokine Assays

Serum IL-1β levels were determined using a commercially rat IL-1β ELISA kit (Invitrogen, BMS630) following the manufacturer’s instructions. Levels of several other cytokines (tumor necrosis factor-α (TNF-α), Interleukin-6 (IL-6), IL-10, IL-18 and CCL5/RANTES), were simultaneously assayed in rat sera with the Luminex technology using the Bio-Plex Pro Rat cytokine kit (Bio-Rad Laboratories) in accordance with the manufacturer’s instructions.

### Measurement of Pituitary and Hypothalamic Fatty Acid Concentrations

Hemi-pituitaries and hemi-hypothalamus were mixed with 800 µl boron trifluoride in methanol (14%) and internal standard (heptadecanoic acid, 10 mg/ml). The mixture was heated at 100°C for 40 min and cooled down to room temperature. Aliquots of 400 µl of heptane and 800 µl of H_2_O were added, centrifuged at 2 000 g for 2 min and methyl esters were then extracted from the upper heptane phase. The sample was concentrated under nitrogen, 100 µl of heptane was added and submitted to gas chromatography analysis. Fatty acid methyl esters (FAME) were analyzed on GC-MS instruments. One microliter of each sample was injected into the instrument with 1/100 split, a Thermo interfaced with a Focus DSQ mass selective detector and equipped with the Xcalibur software package. The mass spectra and retention indices registered in the FAME GC-MS library were obtained using the Thermo FOCUS DSQ Single Quadrupole GC-MS. This was done using the SLB^®^-5ms and the Supelcowax-10 columns made by Supelco (Sigma, France).

### Statistical Analyses

All data were analyzed on GraphPad Prism 6. When data showed normal distribution, as assessed by d’Agostino & Pearson test, statistical differences were determined using one-way ANOVA followed by a Tuckey test. Comparisons between groups were performed using Kruskal-Wallis followed by Dunnett’s test when the data distribution was not normal. Results were considered statistically significant when P ≤ 0.05.

## Results

### Metabolic Disorders Induced by High-Fat Diets in Male Rats

**Figure 1:**
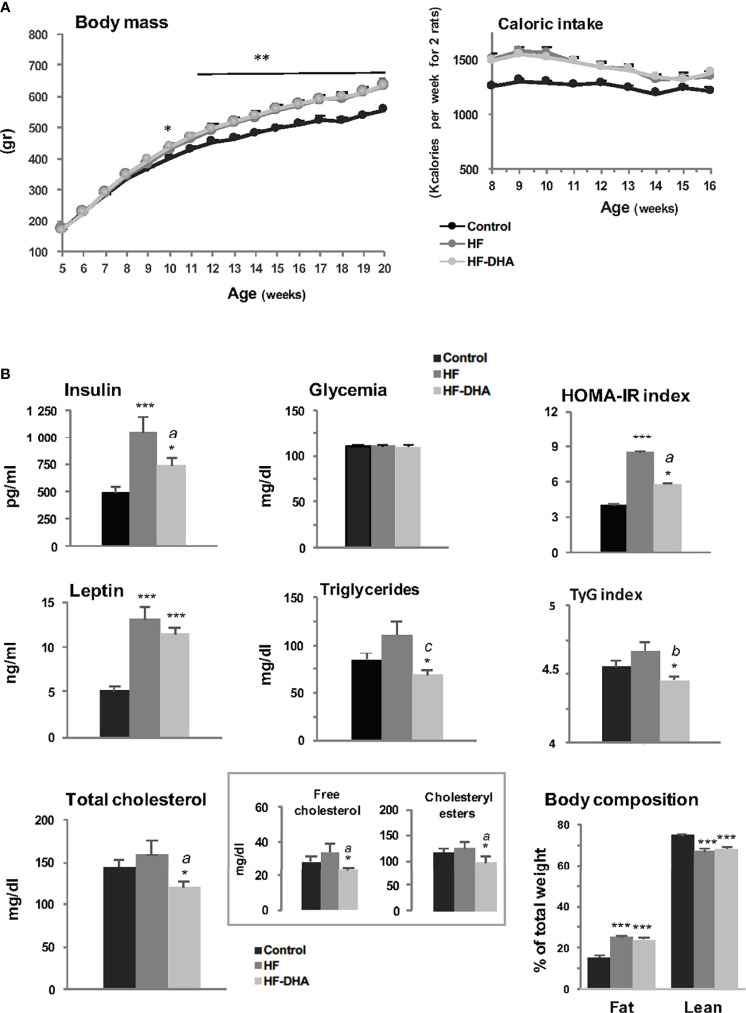
Metabolic status of male rats fed high-fat diets. **(A)** Body mass and caloric intake of male Wistar rats fed control (8.5% kcal from fat) or high-fat (45% kcal from fat) diets supplemented (HF-DHA) or not (HF) with 5% DHA. **(B)** Fasting plasma levels of insulin, leptin, glucose, triglycerides and cholesterol (total, free and esterified forms) and body composition (% fat and lean mass) were measured after 11 weeks of diets. The body composition was measured with the Echo MRI™ system. HOMA-IR and TyG indexes were calculated as a reflect of insulin resistance. Data are expressed as means ± SEM (n= 12 rats) and were analyzed with one-way ANOVA followed by Tukey’s multiple comparison test. *P ≤ 0.05; **P ≤ 0.01; ***P ≤ 0.001 *vs* control. *^a^P ≤* 0.05; *^b^P ≤* 0.01; *^c^P ≤* 0.001 between HF and HF-DHA.

Five-week-old male Wistar rats were fed experimental diets: a regular control diet containing 8% of kcal from fat or two isocaloric high-fat diets, both containing 45% of kcal from fat, that were enriched (HF-DHA) or not (HF) with 5% DHA. As illustrated in **Figure 1A**, rats fed with both high-fat diets were significantly heavier than controls at the end of the study, with an average weight gain of 16%. The difference in body weight gain was already significant after 6 weeks. No difference was observed between HF and HF-DHA diets (**Figure 1A**). Accordingly, caloric intake was identical among rats fed both high-fat diets and higher than in control rats (**Figure 1A**). The body fat mass, determined by magnetic resonance imaging, was significantly higher in the two high-fat groups while the lean body mass was reduced as compared with controls (**Figure 1B**). Plasma insulin was significantly elevated after 11 weeks in rats fed the HF diet. Glycemia was equivalent and thus, the HOMA-IR index, reflecting insulin resistance, was also significantly higher (**Figure 1B**). Leptin levels were significantly increased in HF rats as compared to controls, in parallel with the increase of adiposity. Altogether, this indicates that a resistance to insulin and leptin probably takes place in rats following chronic ingestion of high-fat diets, as already reported (39–41). Circulating triglyceride and cholesterol levels were not significantly elevated and this may be related to their increased hepatic contents (**Supplementary Figure 1**). Supplementation of the HF diet with DHA significantly reduced plasma insulin levels as well as the HOMA-IR index as compared to those in HF rats. It also significantly reduced plasma triglyceride and cholesterol (free as well as esterified forms) levels leading to a significant reduction of the TyG Index, another index of insulin resistance. Leptin levels were not significantly reduced as compared to levels in HF rats, but a significant reduction was attained when increasing the percentage of DHA to 15%, showing that the beneficial effect of DHA is proportional to its amount in the diet (**Supplementary Figure 2A**). Accordingly, this was also associated with a significant decrease of body fat mass as compared to the one of HF rats (**Supplementary Figure 2B**). Altogether, our results indicate that the HF diet used in this study led to alterations in several metabolic parameters in male rats and that supplementation with DHA attenuated most of these alterations, as already reported in rodent models of diet-induced obesity (42, 43). An indirect calorimetry study was performed after 13 weeks of diet, and showed typical effects of high-fat diets on metabolism (**Supplementary Figure 3**). Indeed, both high-fat groups showed a trend toward lower locomotor activity (concomitant with the setting of obesity), increased energy expenditure resulting from higher oxygen consumption (**Supplementary Figures 3A-C**), and a substantial decrease in RER (**Supplementary Figure 3E**) which accounts for higher utilization of lipids as the energy substrate. However, we did not see any differential effect between HF and HF-DHA diets.

### Peripheral and Hypothalamic Inflammation in Rats Fed High-Fat Diets

**Figure 2:**
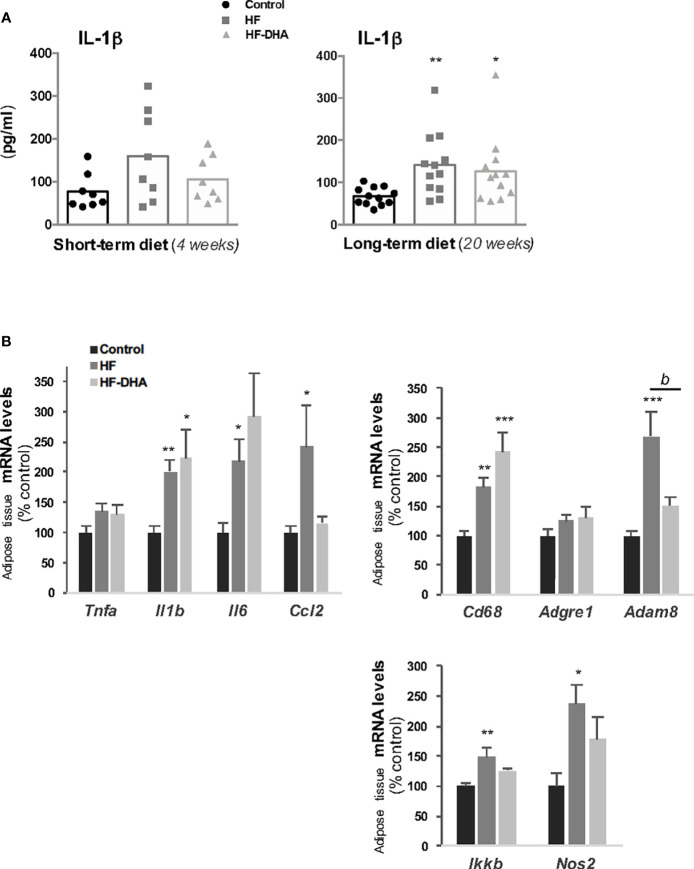
Characterization of peripheral inflammation in rats after 20 weeks of high-fat diets. **(A)** Circulating levels of the pro-inflammatory cytokine interleukin-1β (IL-1β) in rats fed control, HF or HF-DHA diets for 4 and 20 weeks. Levels of IL-1β were assayed by ELISA as described in *Materials and Methods.* The bar height reflects the mean and symbols reflect individual animals. **(B)** Transcript levels of different inflammatory markers in sub-cutaneous adipose tissue after 20 weeks of control or HF diets. Transcript levels were determined by real time PCR, relative levels of genes were normalized to the one of *Cyclophilin* and expressed as % of the control group. Data are means ± SEM from 11-12 **(A)** or 8 **(B)** rats and were analyzed by non-parametric one-way ANOVA (Kruskal-Wallis test) followed by Dunnett’s multiple comparison test. *P ≤ 0.05; **P ≤ 0.01; ***P ≤ 0.001 *vs* control. *^b^P ≤* 0.01 between HF and HF-DHA.

Obesity is associated with a chronic low-grade inflammation in peripheral tissues (44, 45). To determine the inflammatory effects potentially induced by the two high-fat diets used in this study, we first measured the circulating levels of the prototypical pro-inflammatory cytokine IL-1β after a short- or long-term diet (**Figure 2A**). The circulating levels of IL-1β were high in rats receiving a short-term HF diet but did no differ significantly from control rats (*p* = 0.08). After 20 weeks of diet, however, IL-1β levels were significantly increased in HF group as compared to controls. A significant increase was also observed in rats fed HF-DHA diet after 20 weeks. Circulating levels of other cytokines, TNF-α, IL-6, IL-10 and IL-18 were undetectable either after 4 or 20 weeks of diet and CCL5/RANTES levels were unchanged whatever the diet (data not shown). We next studied the expression of several inflammation or macrophage marker genes in the adipose tissue. Transcript levels of the cytokines IL-1β, IL-6 and CCL2 (chemokine ligand 2) were up-regulated after 20 weeks of HF diet whereas no significant change could be detected for *Tnfa* transcript levels. We also measured macrophage genes such as *Cd68*, coding a glycoprotein expressed specifically in macrophages and macrophage-related cells (46); *Adgre1*, encoding the classical macrophage-restricted surface glycoprotein F4/80 (47) and *Adam8* encoding a membrane-anchored protein strongly expressed in monocyte lineage (48). Among the three genes, the expression of both *Cd68* and *Adam8* was significantly increased after the HF diet (**Figure 2B**), suggesting that some immune cell infiltration takes place in adipose tissue. An increase in transcript levels of two inflammation-related factors, IKKβ, playing an essential role in the NF-kappa-B signaling pathway, and the nitric oxide synthase type 2 (NOS2), was also observed in adipose tissue of rats fed the HF diet. Among the seven transcripts that were up-regulated in this tissue, only two of them, *Ccl2* and *Adam8*, had their levels significantly reduced by DHA enrichment of the HF diet (**Figure 2B**). Systemic and adipose tissue inflammation was thus induced in male rats after a long-term HF diet.

**Figure 3:**
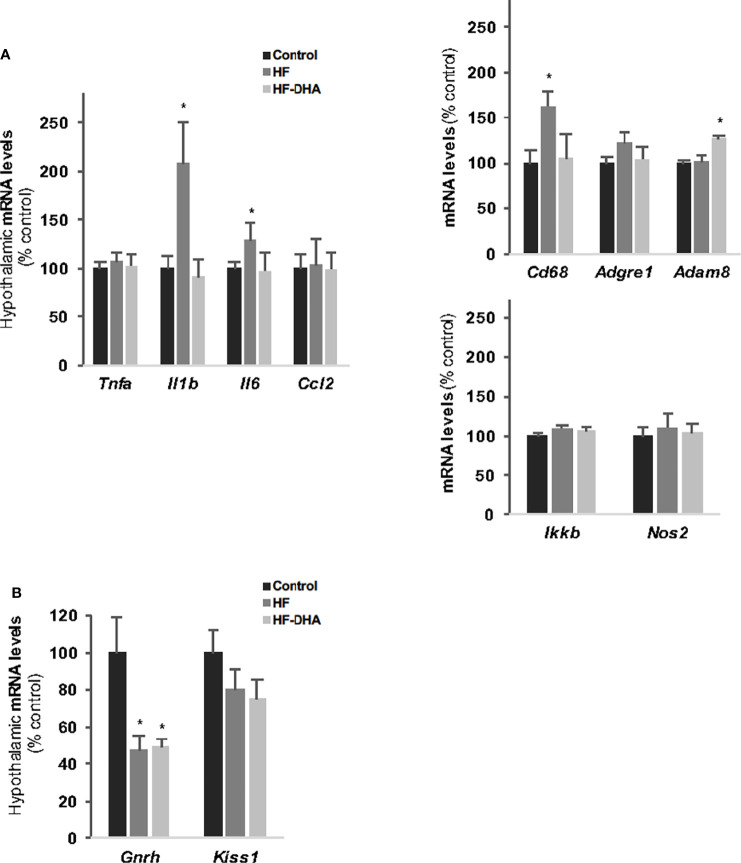
Hypothalamic inflammation and levels of GnRH and Kiss-1 transcripts in rats after 4 weeks of high-fat diets. **(A)** Transcript levels of pro-inflammatory cytokines (*Tnfa*, *Il1b*, *Il6, Ccl2)*, inflammatory-related (*Ikkb* and *Nos2*) and macrophage (*Cd38*, *Adgre1*, *Adam8*) genes in hypothalamus of rats fed control, HF or HF-DHA diets for 4 weeks. **(B)** Hypothalamic transcript levels of *Gnrh* and *Kiss 1* in control and in rats fed short-term diet. Transcript levels were determined and expressed as indicated in **Figure 2B**. Data are means ± SEM (n= 6-8 rats) and were analyzed with Kruskal-Wallis test followed by Dunnett’s multiple comparison test. *P ≤ 0.05 *vs* control group.

Inflammatory pathways have been reported to be activated also in the hypothalamus in rodent models of diet-induced obesity (18, 19), with a shorter time-scale than in periphery (21). We thus determined the hypothalamic levels of inflammatory transcripts in rats fed the high-fat diets for 4 weeks. As shown in **Figure 3A**, the levels of *Il1b*, *Il6* and *Cd68* transcripts were significantly increased in HF rats as compared to those in controls, indicating that inflammation has developed in the hypothalamus. Interestingly, none of these increases could be observed when the HF diet was enriched with DHA. We also determined the gene expression levels of two hypothalamic peptides essential for the neuroendocrine control of reproduction *i.e.* GnRH and Kiss-1 (**Figure 3B**). The mRNA levels of the neurohormone GnRH were markedly reduced in hypothalamus of rats fed high-fat diets (by 47.7% ± 8.1% and 49.6% ± 4% for HF and HF-DHA, respectively). This was not associated with a significant decrease in transcript levels of Kiss1, the main regulator of GnRH secretion (**Figure 3B**). Altogether these data reveal an alteration of gonadotrope activity.

### Absence of Pituitary Inflammation in Rats Fed High-Fat Diets

**Figure 4:**
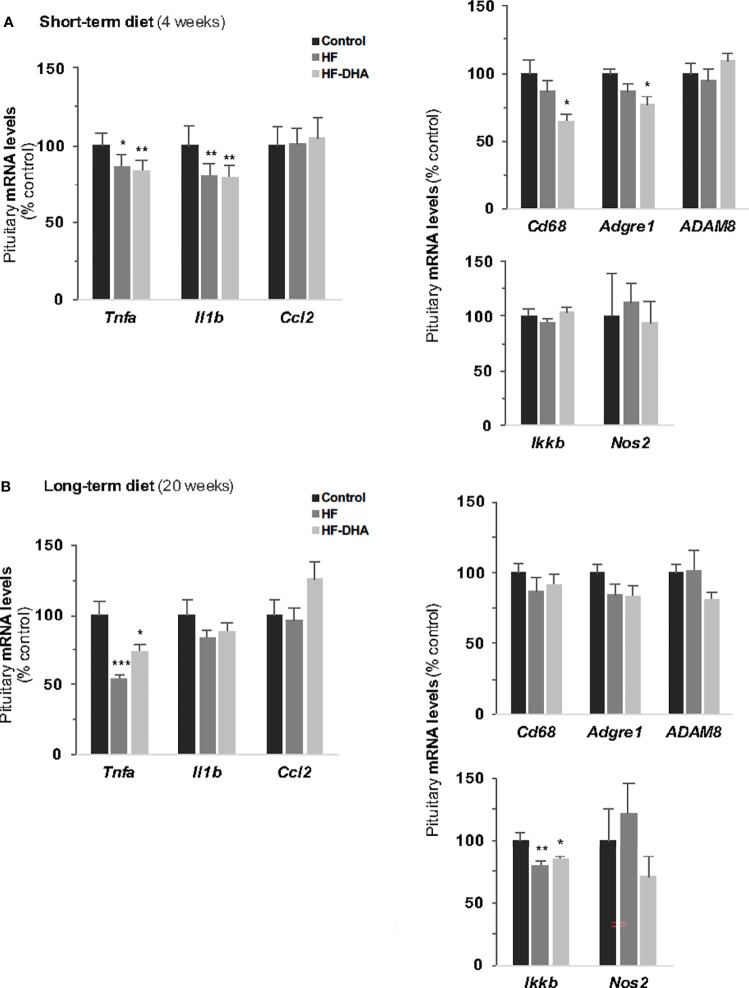
Pituitary inflammation in rats after short-term or long-term high-fat diets. Transcript levels of pro-inflammatory cytokines (*Tnfa*, *Il1b*, *Il6, Ccl2)*, inflammation-related (*Ikkb* and *Nos2*) and macrophage (*Cd38*, *Adgre1*, *Adam8*) genes in pituitaries of rats fed control, HF or HF-DHA diets for 4 **(A)** or 20 weeks **(B)**. Transcript levels were determined and expressed as indicated in **Figure 2B**. Data are expressed as means ± SEM (n= 6-8 rats) and were analyzed with Kruskal-Wallis test followed by Dunnett’s multiple comparison test. *P ≤ 0.05; **P ≤ 0.01; ***P ≤ 0.001 *vs* respective control.

To determine whether the diet-induced inflammation detected in adipose tissue and hypothalamus could also be detected in the pituitary, we performed PCR analysis of the same set of genes in pituitaries of rats after short- and long-term diets. Whatever the gene studied, no significant elevation of transcript levels could be found in pituitaries of rats fed HF after either 4 or 20 weeks (**Figures 4A, B**). We also did not detect any significant increases in rats fed HF-DHA diets. Two additional inflammatory marker genes were analyzed: *Ccl5* and *Ptgs2*, encoding the pro-inflammatory enzyme cyclooxygenase 2 (COX-2), and their expression was unchanged whatever the diet (data not shown). Surprisingly, diet-induced obesity was even associated with a decrease in the level of several pituitary inflammatory markers. The levels of *Tnfa* and *Il1b* transcripts were indeed significantly reduced in HF rats after 4 weeks (**Figure 4A**) and reduction was still observed after 20 weeks for *Tnfa* (**Figure 4B**). Furthermore, the level of *Ikkb* transcripts was also reduced after 20 weeks. The general profile of transcripts was not modified after enrichment of the HF diet with DHA except for *Cd68* and *Adgre1* whose levels after 4 weeks were further decreased, becoming significantly reduced as compared to levels in control rats (**Figure 4A**). Altogether, our results illustrate that the inflammatory response induced by high-fat diets is different between the pituitary and the hypothalamus.

### Differential FA Accumulation and FA-Linked Gene Expression in Pituitary and Hypothalamus of Rats Fed High-Fat Diets

**Figure 5:**
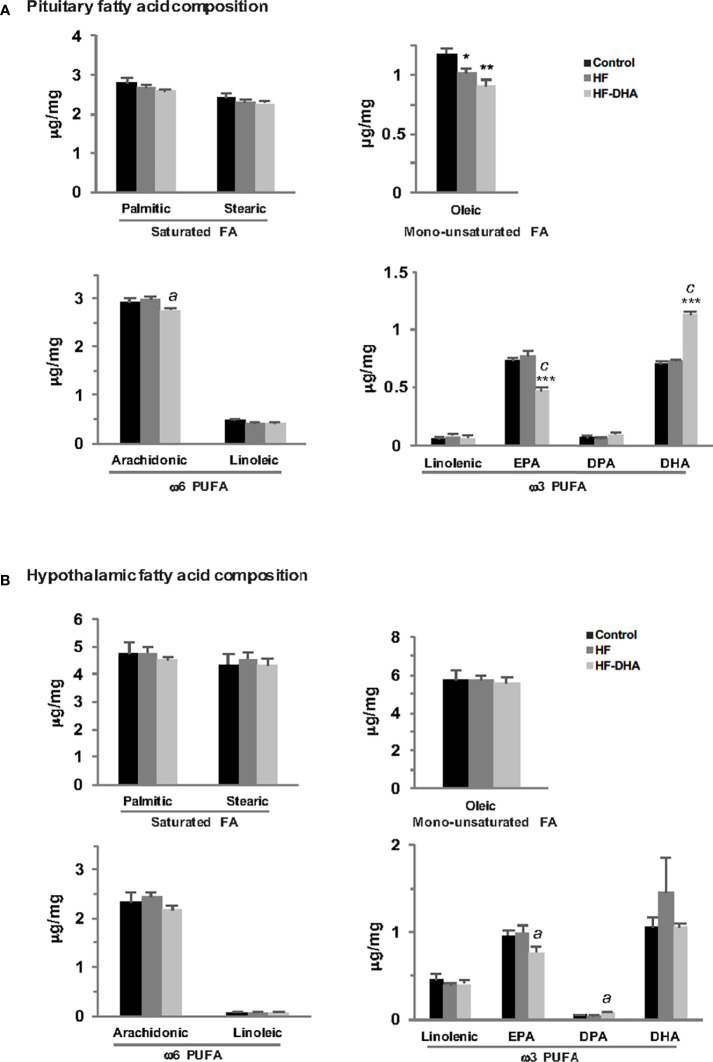
Fatty acid composition in anterior pituitaries and hypothalamus of rats fed short-term high-fat diets. The concentration of saturated FA (palmitic and stearic acids), monounsaturated FA (oleic acid), ω6 PUFA (linoleic, arachidonic acids) and ω3 PUFA (linolenic acid, eicosapentaenoic acid (EPA), docosapentaenoic acid (DPA) and docosahexaenoic acid (DHA) were determined by GCMS as described in *Materials and Methods* in pituitary **(A)** and hypothalamus **(B)** of rats fed control or high-fat diets for 4 weeks. Results are expressed in μg/mg of tissue and are the mean ± SEM of 5 rats in each group. *P ≤ 0.05; **P ≤ 0.01; ***P ≤ 0.001 compared with control group.; *^a^P ≤* 0.05; *^c^P ≤* 0.001 between HF and HF-DHA groups.

Several reports have indicated that the FA composition of the diet can influence FA content in different parts of the brain including the hypothalamus (49, 50). Because saturated FA can induce cytokine expression within the rat hypothalamus (20), we wondered whether the absence of pituitary inflammation could be due to some difference in FA accumulation between both tissues. We thus quantified by GCMS analysis the hypothalamic and pituitary FA composition in rats fed HF and HF-DHA for 4 weeks (**Figure 5**). Different classes of FA were quantified in both tissues: saturated FA (palmitate and stearate), monounsaturated FA (oleate), ω6 PUFA (linoleate, arachidonate) and ω3 PUFA (linolenate, eicosapentaenoate (EPA), docosapentaenoate (DPA) and DHA). We did not detect significant changes in the concentration of saturated, monounsaturated and ω6 PUFA in hypothalamus of HF and HF-DHA rats (**Figure 5**). Similarly, no major change could be detected in the pituitary except for oleic acid whose concentration was significantly decreased by both diets and arachidonic acid displaying a small decrease in HF-DHA rats. Altogether, these data show that there was no major difference in FA accumulation in response to HF diet between the two tissues. Enrichment of HF diet with DHA led to a significant decrease in the concentration of EPA, the precursor of DHA, in both tissues (**Figure 5**). In contrast, we observed a significant increase (1.62 fold over control rats) in pituitary DHA concentrations in rats fed HF-DHA, whereas no change occurred in the hypothalamus. In this latter tissue, the enrichment with DHA rather caused an increase of another ω3 PUFA, the DPA (**Figure 5**).

We also measured the levels of transcripts coding different proteins involved in FA transport and metabolism or in the inflammatory effects of saturated FA such as those coding TLR2 and 4. Three main differences in HF diet-induced changes in gene expression were observed between the pituitary and the hypothalamus. The levels of *Mlycd* transcripts coding Malonyl-CoA decarboxylase, were significantly decreased as compared to control levels only in the pituitary of rats fed HF diet (**Supplementary Figure 4**). The levels of *Tlr4* transcript were significantly increased in rats fed HF diet in the hypothalamus only, suggesting that activation of the inflammatory pathway by saturated FA may be enhanced in the hypothalamus under this diet regimen. HF diet also increased gene expression of the long-chain acyl-coenzyme A synthetase 4 (*Acsl4*) in the hypothalamus but not in pituitary. This latter observation associated with the higher expression of the lipid transporters *Fabp7* and *Fatp1* in hypothalamus suggest an increased FA uptake and conjugation with coenzyme A in the hypothalamus of HF rats as compared to the pituitary. The enrichment of the HF diet with DHA prevented the increase in hypothalamic *Tlr4* transcripts (**Supplementary Figure 4**), as observed for hypothalamic cytokine *Il1b* and *Il6* transcripts (**Figure 3A**) and also decreased the expression of the FA transporters *Fabp7* and *Fatp1*. Altogether, these data reveal different FA sensing and metabolism between the pituitary and the hypothalamus of rats fed high-fat diets.

### High-Fat Diets Decrease Pituitary Gonadotrope Activity

**Figure 6:**
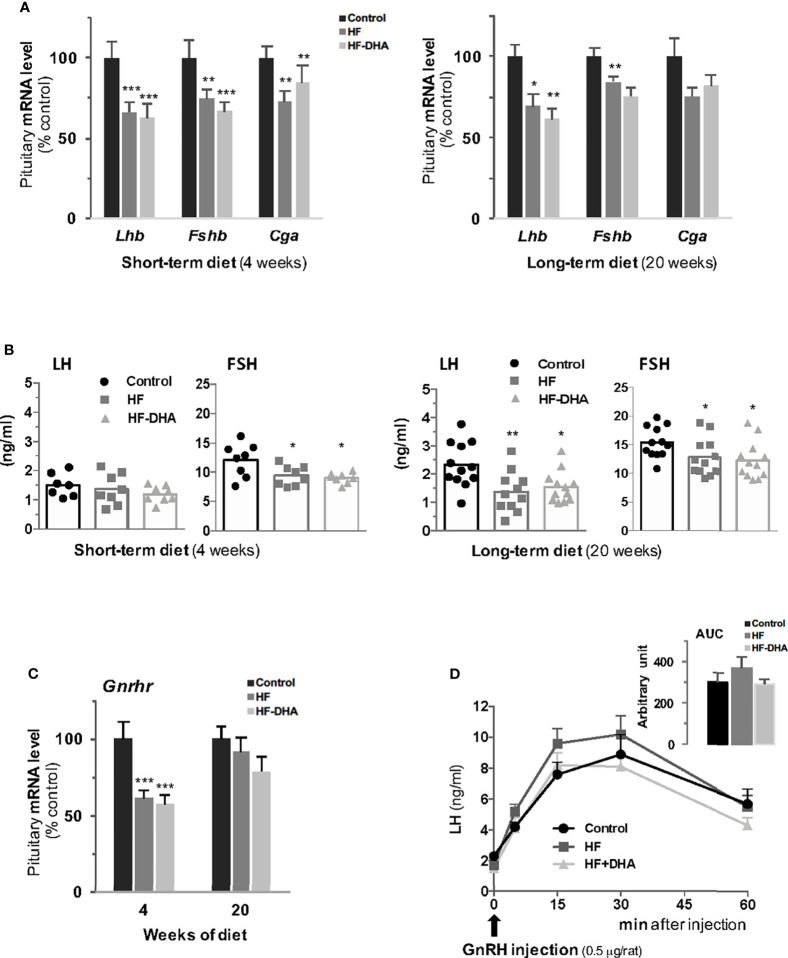
Characterization of pituitary gonadotrope function in rats after short-term or long-term high-fat diets. **(A)** Transcript levels of gonadotropin subunits, *Lhb*, *Fshb* and *Cga*, were measured in pituitaries of rats fed control, HF or HF-DHA diets for 4 or 20 weeks. Transcript levels were determined and expressed as indicated in **Figure 2**. **(B)** The levels of the two circulating gonadotropins, LH and FSH, were simultaneously measured in sera using Luminex technology after 4 and 20 weeks of diet. The bar height reflects the mean. **(C)** Transcript levels of *Gnrhr* in control rats or rats fed high-fat diets for 4 and 20 weeks. **(D)** Pituitary GnRH receptivity in control rats or rats fed high-fat diets for 15 weeks. The natural agonist GnRH (0.5 μg/rat; Sigma LHRH, L 7134, St-Quentin Fallavier, France) was subcutaneously injected and tail blood was collected 0, 5, 15, 30 and 60 min later for LH level determination. The AUC (Area Under the Curve) was calculated for the three groups of rats and no statistical difference was observed. Data are from 6-8 rats in panels A, C and D and from 7-8 (short-term diets) or 11-12 (long-term diets) rats in panel **(B)** Data are expressed as means ± SEM and analyzed with one-way ANOVA followed by Tukey’s multiple comparison test. *P ≤ 0.05; **P ≤ 0.01; ***P ≤ 0.001 compared to control group.

To determine whether the two high-fat diets used in this study induce significant alterations in pituitary gonadotrope activity, we next measured the expression of gonadotropin and GnRH receptor genes as well as the circulating gonadotropin levels after short- and long-term diets. Levels of the three gonadotropin subunit transcripts were significantly decreased in pituitaries of HF rats after 4 weeks and the decrease was maintained after 20 weeks for both gonadotropin β-subunits (**Figure 6A**). The decrease in gonadotropin gene expression was associated with a decrease in their circulating concentrations that was detected after 4 weeks of diet for FSH (**Figure 6B**). The effect of the diet on FSH release cannot probably be attributed to alterations of the local activin/follistatin system, which is a main regulator of FSH (51). Indeed, we did not detect any changes in the expression of the pituitary genes encoding the βA and βB subunits of activin (*Inhba*, *Inhbb*) or follistatin (*Fst*, **Supplementary Figure 5**). After 20 weeks, both gonadotropin concentrations were significantly decreased as compared to those in control rats (**Figure 6B**). There was no significant difference in the effects of the two high-fat diets (**Figures 6A, B**), indicating that the DHA enrichment does not prevent diet-induced disruption of gonadotropin secretion. The levels of *Gnrhr* transcripts were significantly reduced after 4 weeks in pituitaries of rats fed HF or HF-DHA but returned to control values after 20 weeks (**Figure 6C**). The LH response to GnRH injection was also determined in rats after a long-term diet. As illustrated in **Figure 6D**, the net (AUC) LH responses did not differ between rats fed control, HF or HF-DHA diets, in line with the unchanged levels of *Gnrhr* transcripts at 20 weeks. Altogether, these results indicate that LH responsiveness to GnRH may not be altered by long-term high-fat diets.

**Figure 7:**
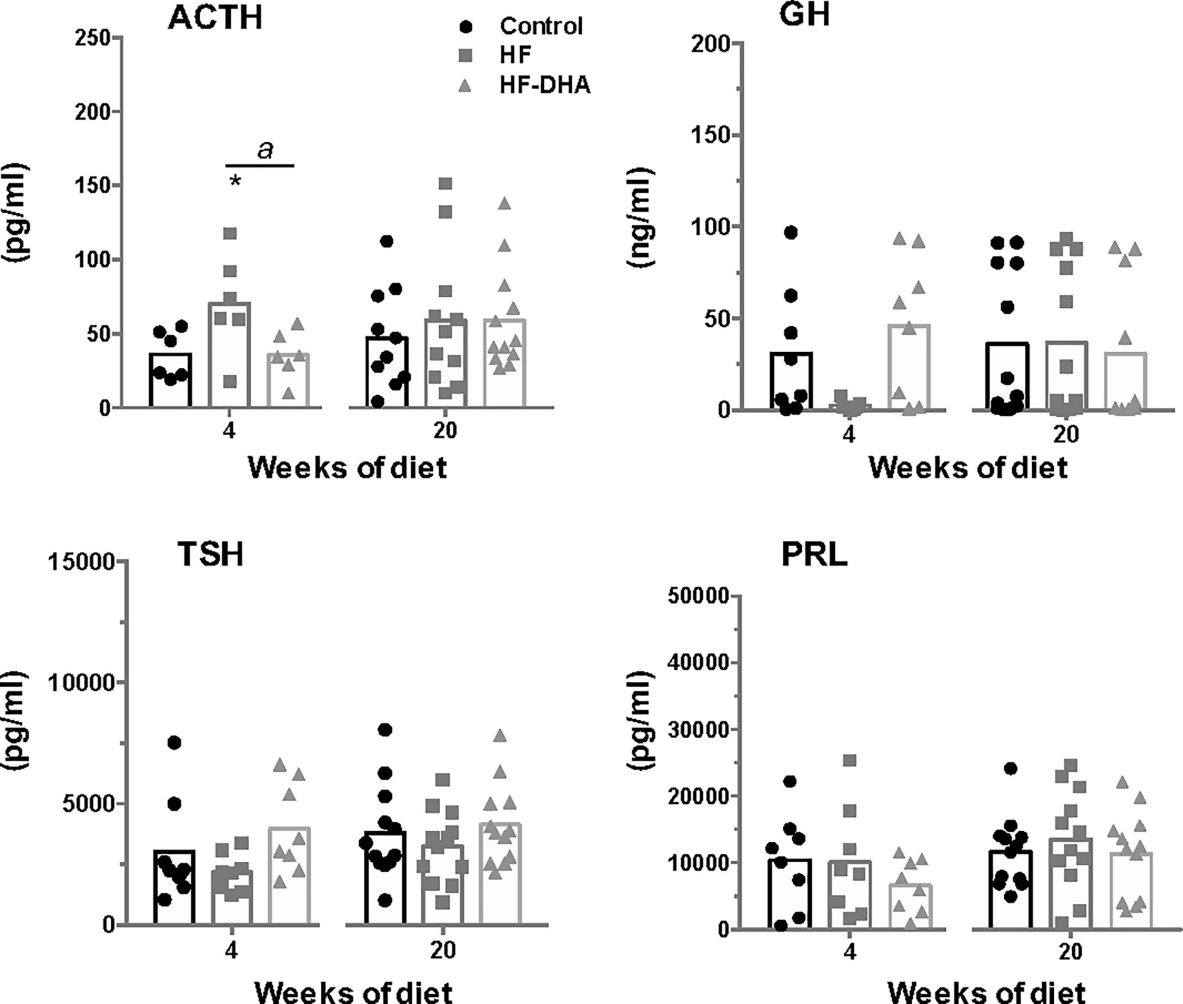
Circulating levels of non-gonadotrope pituitary hormones rats after short-term or long-term high-fat diets. Serum levels of the pituitary hormones, ACTH, GH, PRL and TSH, were measured after 4 and 20 weeks of diet as described in *Materials and Methods*. The bar height reflects the mean. Data are expressed as means ± SEM (n= 6-8 and n=10-12 rats for short- and long-term diets, respectively) and were analyzed with Kruskal-Wallis test followed by Dunnett’s multiple comparison test. *P ≤ 0.05 *vs* control. *^a^P ≤* 0.05 between HF and HF-DHA.

Because obesity has been correlated with dysregulation of multiple pituitary hormones (52), we also investigated whether non-gonadotrope endocrine cells were affected by the HF and HF-DHA diets (**Figure 7**). A short-term HF diet significantly increased circulating ACTH levels as previously reported (53, 54). On the contrary, the levels of GH appeared reduced in HF rats after 4 weeks, as already reported in animal models of diet-induced obesity (55, 56), albeit the observed decrease was not statistically different due to the high interindividual variability. Similarly, circulating TSH levels were not statistically decreased in HF rats as compared to controls. In rats fed HF-DHA, the circulating values of the three pituitary hormones were similar than the ones in control rats. PRL levels were unaffected whatever the diet regimen. After 20 weeks of diet, the circulating levels of all four pituitary hormones in obese HF and HF-DHA rats were similar that controls (**Figure 7**). Altogether, these results reveal a differential susceptibility to high-fat diets among the different endocrine cell types of the pituitary.

### Alteration of Testicular Endocrine Activity in Rats Fed High-Fat Diets

**Figure 8:**
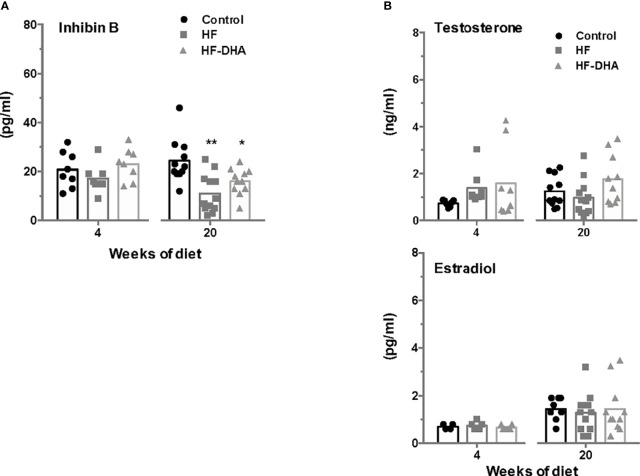
Circulating inhibin B and sex steroid levels in rats after short-term or long-term high-fat diets. Serum levels of inhibin B **(A)** and sex steroids, testosterone and estradiol **(B)** were determined after 4 and 20 weeks of diet, as described in *Materials and Methods*. The bar height reflects the mean. Data are expressed as means ± SEM from 7-8 and 10-12 rats for short- and long-term diets, respectively and were analyzed by one-way ANOVA followed by non-parametric one-way ANOVA (Kruskal-Wallis test) followed by Dunnett’s multiple comparison test. *P ≤ 0.05; **P ≤ 0.01 *vs* control.

To explore whether the observed down regulation of gonadotropin expression in animals fed short- and long-term high-fat diets could be due to alterations in testicular feedbacks, we next measured inhibin B and sex steroid levels in rats after 4 and 20 weeks of diet (**Figure 8**). HF rats exhibited a significant decline of circulating inhibin B levels after 20 weeks of regimen. At this time, DHA enrichment did not restore inhibin B levels. The circulating levels of testosterone were not significantly modified by either HF or HF-DHA diet, whatever the duration of the diet, and not different between the two diets. Similarly, the circulating levels of estradiol were unaffected by both high-fat diets at 4 and 20 weeks of the regimen (**Figure 8**).

## Discussion

It has been clearly demonstrated that overnutrition causes an activation of inflammatory pathways in several metabolically linked tissues. In addition, more recent evidence indicates that an inflammation is established earlier in the hypothalamus and disrupts the central control of energy homeostasis (19). Although FA can be sensed by the pituitary and alter the synthesis of gonadotropins (7, 30), the possible contribution of a pituitary inflammation in the reported alterations in the hypothalamic-pituitary control of reproduction in a number of obese patients and in animal models of diet-induced obesity, has never been addressed.

In the present report, we provide experimental data showing that after only four weeks, the HF diet caused significant alterations in gonadotropin synthesis and release. We, however, did not detect any concomitant increase in the gene expression of inflammatory cytokines or mediators such as Ikkβ and NOS2 within the pituitary although an increased expression of cytokine or macrophage genes was detected in the hypothalamus of male rats, in agreement with previous reports (19, 21, 22, 25, 57). As previously reported (15, 18, 21), increases of hypothalamic cytokine transcripts were rather modest and only a subset of inflammatory markers exhibited significant changes. In agreement with this, the time course analysis of inflammatory markers in rats fed HF diet for 28 days, revealed that cytokines only exhibited temporary increases in their expression with distinct temporal pattern (21). The mechanisms explaining how such small and fluctuating elevations of hypothalamic cytokines can disrupt the control of energy balance remains to be fully elucidated. After a longer period of overnutrition, when several of the inflammatory markers were found to be increased in both blood and adipose tissue, no such increase was present in the pituitary although gonadotropin secretion was still altered. Accordingly, our experimental data also support the idea that macrophages do not infiltrate the pituitary since no change in the expression of macrophage markers was detected in contrast to what we observed in the hypothalamus. Altogether, our data suggest that the pituitary may be less sensitive to inflammation than hypothalamus or metabolically linked peripheral tissues. The mechanisms explaining the absence of pituitary inflammation remain to be determined. Several studies have reported accumulation of saturated FA in the hypothalamus when dietary saturated FA are consumed in excess (49, 50). Because saturated FA are potential triggers of tissue inflammation, this led us to speculate that a differential accumulation of saturated FA between the hypothalamus and the pituitary may explain their differential inflammation. However, GCMS determinations of hypothalamic and pituitary FA concentrations after a short-term HF diet do not support such hypothesis. Indeed, we did not detect significant increases in the concentrations of saturated FA, palmitic and stearic acids, in neither of the two organs. Another explanation may be the differential regulation of FA action or metabolism. Indeed, we report here that the gene expression of TLR4, mediating saturated FA-induced production of cytokines, was up-regulated by the HF diet in the hypothalamus but not the pituitary. Further, supporting the possible involvement of *Tlr4* in mediating cytokine induction, we report here that DHA enrichment of the diet, which prevented hypothalamic increase of cytokines also prevented the increase of *Tlr4*. We also found a decrease in the gene expression of malonyl-CoA decarboxylase in pituitaries but not hypothalamus of rats fed HF diet. Interestingly, inactivation of malonyl-CoA decarboxylase was recently shown to attenuate the inflammatory response induced by treatment with LPS in neonatal cardiomyocytes and peritoneal macrophages (58). A differential regulation of TLR-4 and malonyl-CoA decarboxylase expression may thus have contributed to protect the pituitary from inflammation. Intriguingly, we showed that rather than an absence of pituitary inflammation, there was even a down-regulation of inflammatory marker expression in the pituitary after both short- and long-term diets. The mechanisms allowing such specific inflammatory response remain to be determined. The pituitary is known to be a major source of α-MSH, a peptide known to have potent anti-inflammatory effects (59). Whether pituitary α-MSH levels are increased under high-fat diets, and whether α-MSH could efficiently counteract pituitary inflammatory response deserve further investigation. Another explanation would be that pituitaries of rats fed high-fat diets produce high levels of pro-resolving mediators, which are involved in the resolution of inflammation (60). The precise characterization of the inflammatory response in the pituitary is however compromised by the cellular heterogeneity of the gland in which the different endocrine lineages were shown here to be differentially regulated by the HF diet. It is possible that inflammation takes place in some but not all pituitary cell types, obscuring potential changes in a specific population when analyzing the whole organ. Among pituitary cells, the folliculo-stellate cells probably play a crucial role in the communication between the immune and endocrine systems. Single cell RNA-sequencing analyses should bring valuable information on the mechanisms underlying the inflammatory response to HF diet within the pituitary, and notably within gonadotrope cells.

We observed in the present study that male rats fed HF diet exhibited altered gonadotrope function as previously reported (12, 13). Analysis of gonadotrope function was performed by measuring both gonadotropin β-subunits and circulating gonadotropin levels not only after a long-term exposure as done in most previously published studies but also after only 4 weeks of HF diet, thus before the development of a significative weight gain in rats. Our data showed that the expression of both gonadotropin specific subunits was already altered at this early time of the regimen. Concurrent determinations of serum gonadotropin concentrations showed that only FSH concentrations were significantly reduced. This may be explained by the fact that FSH appears to be released mostly through the constitutive pathway in accordance to its rate of synthesis while LH is mostly released through the regulated pathway (61). At this early stage of the regimen, our data on circulating cytokines and pituitary inflammatory transcripts do not support a role of systemic or local inflammation in the observed decrease in gonadotropin secretion. The decrease seems no more due to increased inhibitory feedbacks from testes as there were no changes in circulating inhibin B or testosterone, suggesting that the HF diet operated at the level of the hypothalamic-pituitary complex. Supporting the hypothesis of an alteration of the neuroendocrine control of reproduction, we report here a decreased expression of the neuropeptide GnRH. At this stage of the regimen, we provide evidence that an inflammatory response has already been established in the hypothalamus. The hypothalamic insulin resistance known to be induced by inflammation may explain the observed decrease in GnRH and gonadotropin expression. Indeed, several experiments support a crucial role of brain insulin signaling in the control of reproduction. Among these is the demonstration that neuron-specific deletion of the insulin receptor in mice led to hypothalamic hypogonadism with decreased circulating LH levels in both transgenic males and females (62). Because leptin is also a metabolic regulator of the reproductive brain (63, 64), the development of cerebral leptin resistance due to inflammation could also have contributed to the decreased gonadotrope activity in rats fed short-term HF diet. Leptin receptor is expressed by Kiss-1 neurons and Kiss-1 mRNA levels have been reported to be significantly diminished in leptin deficient *ob/ob* mice, leading to the idea that reproductive deficits associated with leptin deficient state may be attributable, in part, to diminished expression of Kiss-1 (65, 66). In the present study, we found that transcript levels of Kiss-1 were not significantly decreased by a short-term HF diet. Such discrepancy may be explained by the fact that HF diet is a less drastic model of leptin resistance than genetic leptin deficiency. Indeed, and in agreement with our study in rats, other studies have reported that HF diets failed to induce overt changes in hypothalamic expression of Kiss-1 in C57BL/6J mice (67, 68). It is also possible that a significant decrease in Kiss-1 expression could have been observed in restricted brain regions such as the arcuate nucleus. Our observation of elevated circulating ACTH levels in rats fed HF diet is indicative of an increased CRH release associated with hypothalamic inflammation. Supporting this hypothesis is the fact that intracerebral administration of cytokines has been shown to increase CRH release in rats (69). Because CRH has been implicated in the suppression of reproductive neuroendocrine function in rats and several other mammalian species (70), this could have contributed to the observed decrease in GnRH and gonadotropin synthesis and release. The downward trend in TSH and GH levels that we observed in the present report may also result from hypothalamic inflammation since intracerebral cytokines were shown to decrease GH and TSH secretion (71).

After a longer exposure to HF diet, the gonadotrope activity was still reduced as evidenced by decreases in circulating gonadotropin and pituitary gonadotropin transcript levels. As for the short-term diet, this decrease was not associated with increased testosterone negative feed-back. Inhibin B levels were even significantly lowered at this time, probably reflecting the altered FSH regulation of the Sertoli cells in the testes. As observed for the short-term HF diet, no inflammation has taken place within the pituitary that could have contributed to the alteration of gonadotrope activity. Instead, we hypothesize that enhanced circulating IL-1β levels at this time of the regimen may contribute to the reduced gonadotropin production. Indeed, several studies have reported that basal or stimulated gonadotropin synthesis and release can be altered by IL-1β both *in vivo* and *in vitro* (72, 73). Furthermore, the long-term HF diet also led to metabolic disorders such as increases in circulating insulin and leptin levels, indicative of insulin and leptin resistance, as classically reported in animal models of diet-induced obesity (39, 40, 74). Because insulin and leptin have been shown to directly target the pituitary to increase the expression of gonadotropins (3, 4), alterations of their signaling in the pituitary may have contributed to the observed decrease in gonadotrope activity. Brothers and collaborators have however reported that the pituitary of diet-induced obese female mice retained insulin sensitivity (75). The possibility that insulin signaling may also be maintained in pituitaries of male rats would deserve additional studies. Interestingly, FSH and LH were the only pituitary hormones remaining affected at this time by the regimen, further underlining the differential susceptibility of endocrine lineages to metabolic disorders.

Dietary supplementation with ω3 PUFA can reduce one or more factors of the metabolic syndrome (42, 43) and has been shown to revert diet-induced hypothalamic inflammation (27). We here thus analyzed the effects of a HF diet enriched with 5% of DHA on pituitary gonadotrope activity and inflammation. In our experimental paradigm, DHA accounted for approximately 2.5% of the total energy, which is in the range used in human clinical trials (76). We demonstrate here that DHA enrichment improved the metabolic status of male rats, as previously reported (42, 77), as it normalized several metabolic markers that were increased by the HF diet. Moreover, it also prevented hypothalamic inflammation as all HF diet-induced increases of inflammatory markers were normalized under DHA. The enrichment of diet with DHA was, however, not able to prevent alterations in gonadotrope activity either after a short- or a long-term treatment. This absence of effect cannot be explained by insufficient DHA delivery to the pituitary as we detected an important increase of the DHA content in the pituitary. Because DHA gives rise to anti-inflammatory compounds such as resolvins (78), this may have contributed to reduce inflammation in the pituitary. Interestingly, there was no change in the hypothalamic content of DHA but, instead, we detected a significant increase in the ω3 PUFA, DPA. Recent studies have implicated DPA in the improvement of several metabolic disease markers, including insulin sensitivity (79), and DPA has been shown to display a greater anti-inflammatory effect than EPA or DHA in a model of colorectal cancer (80). Thus, in this tissue, DPA rather that DHA may have reversed inflammatory changes caused by the consumption of the HF diet. Altogether, our study underlines different FA incorporation and metabolism between hypothalamus and pituitary. The decrease in the content of the DHA precursor, EPA, that we observed in both tissues probably reflected an adaptive response to the high supply of dietary DHA.

In summary, the data presented here provide strong evidence of an alteration of gonadotrope activity in male rats after feeding a high-fat diet for 4- and 20-weeks, which occurred without any increase in pituitary gene expression of cytokines (TNFα, IL-1β, CCL2, CCL5), macrophage related markers (CD68, ADGR1, ADAM8) or inflammatory related markers (NOS2, IKKβ, COX-2). Future studies are needed to further characterize the molecular alterations occurring in each pituitary cell type that should help to understand the high susceptibility of reproductive function to metabolic status as well as the differential response of the endocrine cells to nutritional information. Because the inflammatory response induced by high fat diets appears to differ between male and female rodents, a comparative analysis of inflammatory response in both sexes would be of great interest.

## Data Availability Statement

The original contributions presented in the study are included in the article/**Supplementary Material**. Further inquiries can be directed to the corresponding author.

## Ethics Statement

The animal study was reviewed and approved by French Ministry of Research (approval # 4187-2016021715365460).

## Author Contributions

GG conceived, designed and performed the experiments, analyzed primary data, aggregated data and contribute to the writing of the manuscript. CR contributed to *in vivo* experiments, performed metabolic assays and analyzed primary data. DL’H contributed to *in vivo* experiments. NK contributed to PCR analysis. FG performed sex steroid quantification using GC-MS. JD performed fatty acid quantification using GC-MS. PD and PG contributed to inflammation study. CM and CC-G contributed to experimental design and analyzed primary data. JC-T conceived and supervised the project, analyzed aggregated data and wrote the manuscript. All authors contributed to the article and approved the submitted version.

## Funding

This study was supported by grants from ANR-18-IDEX-0001, IdEx Université de Paris 2019, CNRS and Inserm.

## Conflict of Interest

The authors declare that the research was conducted in the absence of any commercial or financial relationships that could be construed as a potential conflict of interest.

## Publisher’s Note

All claims expressed in this article are solely those of the authors and do not necessarily represent those of their affiliated organizations, or those of the publisher, the editors and the reviewers. Any product that may be evaluated in this article, or claim that may be made by its manufacturer, is not guaranteed or endorsed by the publisher.

## Supplementary Material

Supplementary Figure 1 Lipid content in the liver of rats after long-term high-fat diets. Triglycerides and cholesterol (total, free and esterified forms) contents were measured in the liver of rats fed control, HF or HF-DHA diets for 20 weeks. Data are expressed as means ± SEM (n= 12 rats) and were analyzed with one-way ANOVA followed by Tukey’s multiple comparison test. **P ≤* 0.05; ***P ≤* 0.01; ****P ≤* 0.001 compared to control group. ^a^P< 0.05 between HF and HF-DHA groups.

Supplementary Figure 2 Metabolic parameters and body composition of rats fed HF diet supplemented with 15% DHA. **(A)** Fasting plasma levels of insulin, leptin and triglycerides were measured in rat fed control, HF and HF supplemented with 15% DHA for 12 weeks. Data are expressed as means ± SEM (n= 12 rats) and were analyzed with one-way ANOVA followed by Tukey’s multiple comparison test. **P ≤* 0.05; ***P ≤* 0.01; ****P ≤* 0.001 compared to control group. *^b^P ≤* 0.01 between HF and HF-DHA15%. **(B)** The body composition of rats (% fat and lean mass) was measured after 12 weeks of diet. ***P ≤* 0.01; ****P ≤* 0.001 compared to control group. *^b^P ≤* 0.01 between HF and HF-DHA groups.

Supplementary Figure 3 Indirect calorimetry of rats after 13 weeks high-fat diets. Rats were placed in calorimetric cages after 13 weeks of diet to monitor several metabolic parameters: **(A)** Ambulatory activity, **(B)** Energy expenditure, **(C)** Volume of oxygen used, **(D)** Volume of carbon dioxide rejected and **(E)** Respiratory Exchange Ratio (RER; VCO_2_/VO_2_). Data are expressed as means ± SEM (n= 8 rats per group) and analyzed with one-way ANOVA followed by Tukey’s multiple comparison test. **P ≤* 0.05; ***P ≤* 0.01; ****P ≤* 0.001 *vs* respective control group.

Supplementary Figure 4 Expression levels of genes coding proteins involved in the transport, metabolism or action of FA in anterior pituitaries and hypothalamus of rats fed short-term high-fat diets. The transcript levels of several genes were determined in pituitary **(A)** and hypothalamus **(B)** of rats fed short-term HF diets: genes involved in cell transport of FA: *Fabp7*, Fatty acid binding protein 7; *Fatp1*, Fatty acid transport protein 1; *Cd36*, fatty acid transporter/cluster of differentiation 36; *Acsl1*, Acyl-Co1 synthetase long-chain family member 1; *Acsl4*, Acyl-Co1 synthetase long-chain family member 4; genes involved in FA metabolism: *Mlycd*, Malonyl-CoA decarboxylase; *Acc2*, Acetyl-CoA carboxylase 2; *Fasn*, Fatty acid synthase and genes mediating FA inflammatory effects: *Tlr2* and *Tlr4*, Troll-like receptor 2 and 4. Data are expressed as means ± SEM (n= 11-12 rats) and were analyzed with one-way ANOVA followed by Tukey’s multiple comparison test. **P ≤* 0.05; ***P ≤* 0.01compared with control group.; *^a^P ≤* 0.05 between HF and HF-DHA groups.

Supplementary Figure 5 Pituitary transcript levels of βA and βB subunits of activin and follistatin after short-term high-fat diets. Transcript levels of βA and βB subunits of activin (*Inhba*, *Inhbb*) and follistatin (*Fst*) were measured in pituitaries of rats fed control, HF or HF-DHA diets for 4 weeks. Data are expressed as means ± SEM from 7 rats and were analyzed by non-parametric one-way ANOVA (Kruskal-Wallis test) followed by Dunnett’s multiple comparison test. There was no statistical difference between the different groups of rats.

## Abbreviations

ACTH, Adrenocortitropic hormone; ADAM8, ADAM metallopeptidase domain 8; CCL2, C-C motif chemokine ligand 2; CCL5, C-C motif chemokine ligand 5; CD68, Cluster of differentiation 68; CGA, Glycoprotein hormone alpha polypeptide; Cyclooxygenase-2, COX-2; DHA, docosahexaenoic acid; DPA, docosapentaenoic acid; EPA, eicosapentaenoic acid; FA, fatty acid; FBS, fetal bovine serum; FSH, Follicle-stimulating hormone; GC-MS, Gas chromatography-mass spectrometry; GH, Growth hormone; GnRH, gonadotropin-releasing hormone; HF, High fat; HOMA-IR, Homeostasic model assessment for insulin resistance; IL-1β, Interleukin-1 beta; IL-6, Interleukin-6; IKKβ, Inhibitor of nuclear factor kappa-B kinase subunit beta; LH, luteinizing hormone; NOS2, Nitric oxide synthase 2; PRL, Prolactin; PUFA, polyunsaturated FA; TNF-α, Tumor necrosis factor alpha; TSH, Thyroid-stimulating hormone; TyG index, Triglyceride-glucose index.
