# Supplementary figures and images for: Disruption of Pituitary Gonadotrope Activity in Male Rats After Short- or Long-Term High-Fat Diets Is Not Associated With Pituitary Inflammation
Source: Front Endocrinol (Lausanne). 2022 Apr 13;13:877999. doi: 10.3389/fendo.2022.877999 (PMC9043610; doi:10.3389/fendo.2022.877999)

**Supplemental figure 1**

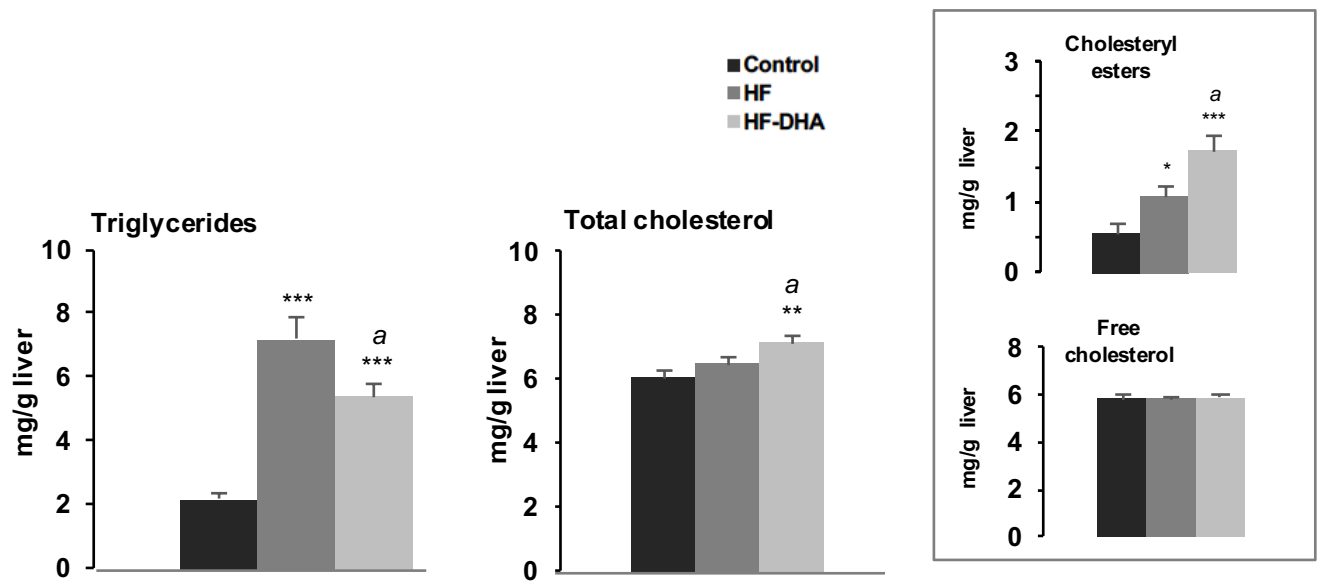

Supplement: Supplementary Figure 1 — Lipid content in the liver of rats after long-term high-fat diets. Triglycerides and cholesterol (total, free and esterified forms) contents were measured in the liver of rats fed control, HF or HF-DHA diets for 20 weeks. Data are expressed as means ± SEM (n= 12 rats) and were analyzed with one-way ANOVA followed by Tukey’s multiple comparison test. *P ≤ 0.05; **P ≤ 0.01; ***P ≤ 0.001 compared to control group. aP< 0.05 between HF and HF-DHA groups. [file DataSheet_1.pdf]

## Supplemental figure 2

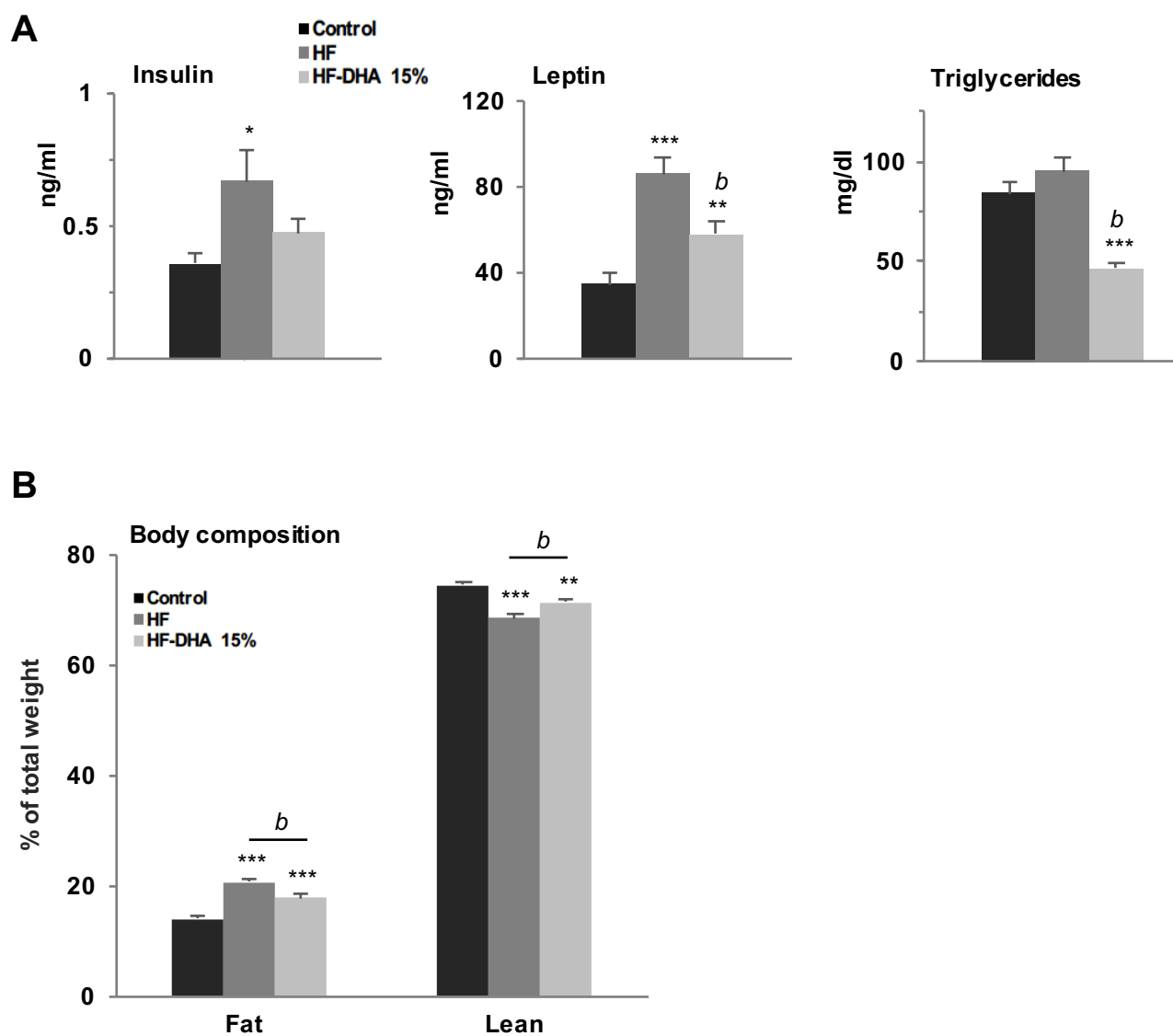

Supplement: Supplementary Figure 2 — Metabolic parameters and body composition of rats fed HF diet supplemented with 15% DHA. (A) Fasting plasma levels of insulin, leptin and triglycerides were measured in rat fed control, HF and HF supplemented with 15% DHA for 12 weeks. Data are expressed as means ± SEM (n= 12 rats) and were analyzed with one-way ANOVA followed by Tukey’s multiple comparison test. *P ≤ 0.05; **P ≤ 0.01; ***P ≤ 0.001 compared to control group. bP ≤ 0.01 between HF and HF-DHA15%. (B) The body composition of rats (% fat and lean mass) was measured after 12 weeks of diet. **P ≤ 0.01; ***P ≤ 0.001 compared to control group. bP ≤ 0.01 between HF and HF-DHA groups. [file DataSheet_2.pdf]

### Supplemental figure 3

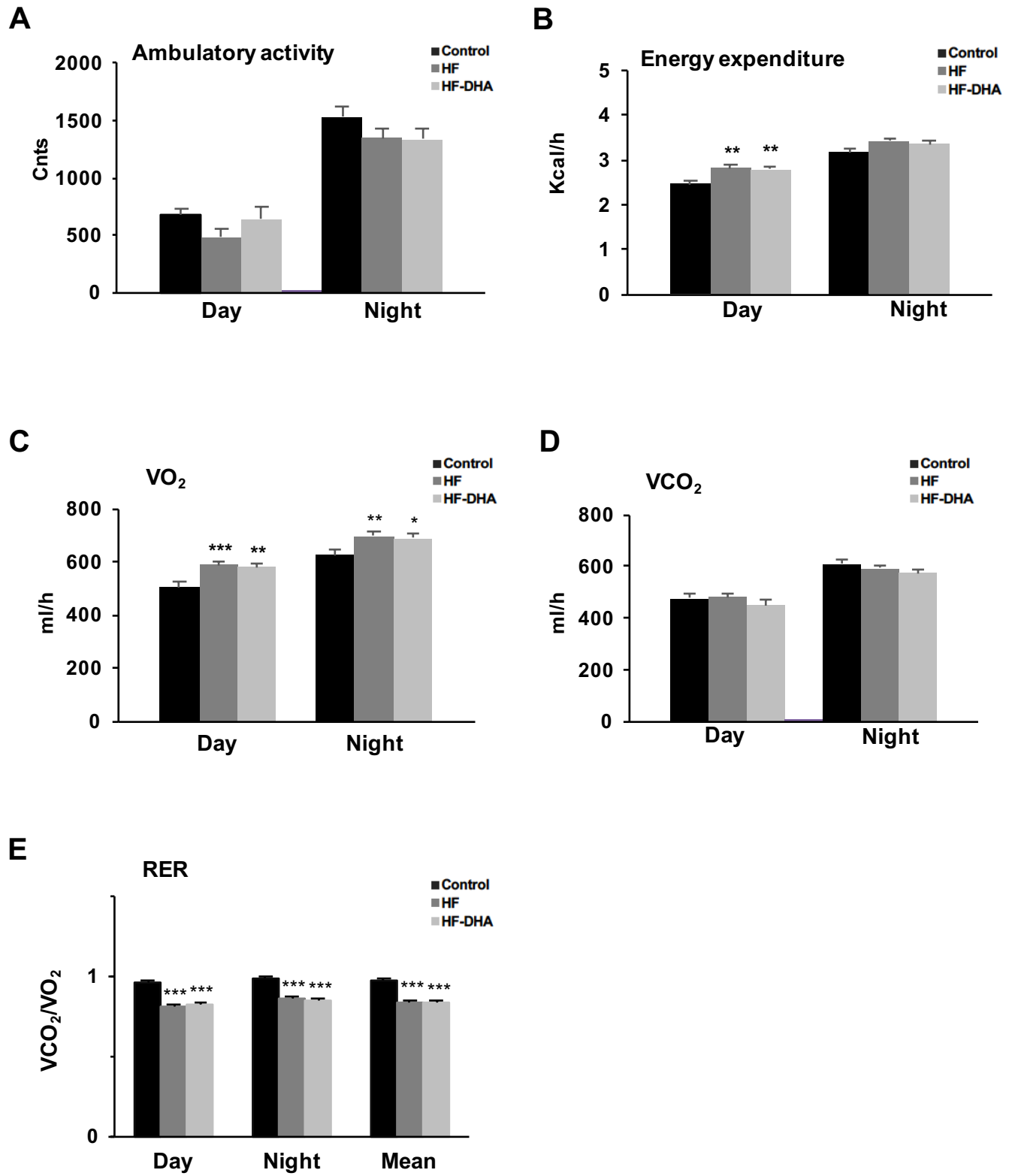

Supplement: Supplementary Figure 3 — Indirect calorimetry of rats after 13 weeks high-fat diets. Rats were placed in calorimetric cages after 13 weeks of diet to monitor several metabolic parameters: (A) Ambulatory activity, (B) Energy expenditure, (C) Volume of oxygen used, (D) Volume of carbon dioxide rejected and (E) Respiratory Exchange Ratio (RER; VCO2/VO2). Data are expressed as means ± SEM (n= 8 rats per group) and analyzed with one-way ANOVA followed by Tukey’s multiple comparison test. *P ≤ 0.05; **P ≤ 0.01; ***P ≤ 0.001 vs respective control group. [file DataSheet_3.pdf]

## Supplemental figure 5

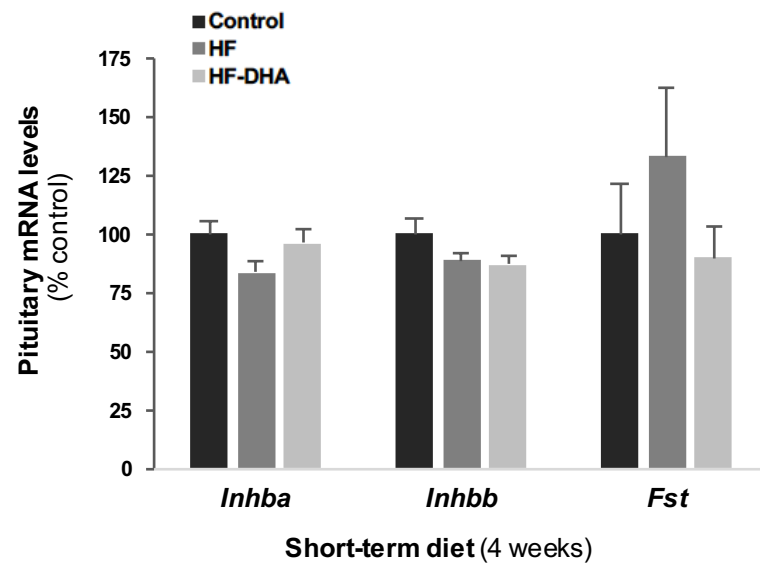

Supplement: Supplementary Figure 5 — Pituitary transcript levels of βA and βB subunits of activin and follistatin after short-term high-fat diets. Transcript levels of βA and βB subunits of activin (Inhba, Inhbb) and follistatin (Fst) were measured in pituitaries of rats fed control, HF or HF-DHA diets for 4 weeks. Data are expressed as means ± SEM from 7 rats and were analyzed by non-parametric one-way ANOVA (Kruskal-Wallis test) followed by Dunnett’s multiple comparison test. There was no statistical difference between the different groups of rats. [file DataSheet_5.pdf]
