# Supplementary material for: Disruption of Pituitary Gonadotrope Activity in Male Rats After Short- or Long-Term High-Fat Diets Is Not Associated With Pituitary Inflammation
Source: Front Endocrinol (Lausanne). 2022 Apr 13;13:877999. doi: 10.3389/fendo.2022.877999 (PMC9043610; doi:10.3389/fendo.2022.877999)

Supplemental figure 4

A

Pituitary gene expression, **short-term diet (4 weeks)**

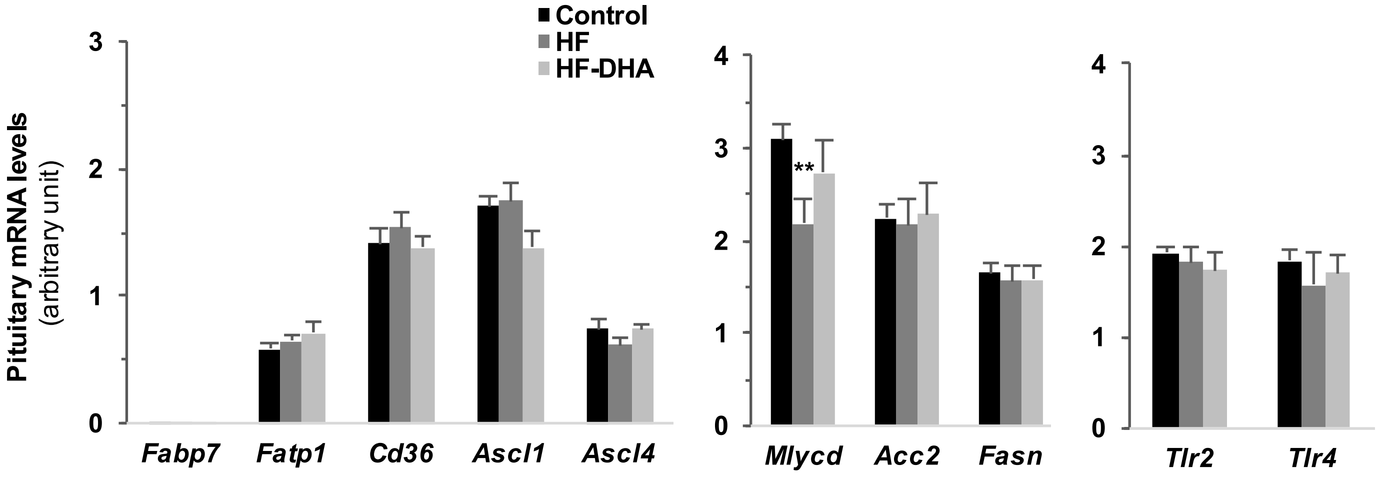

B

Hypothalamic gene expression, **short-term diet (4 weeks)**

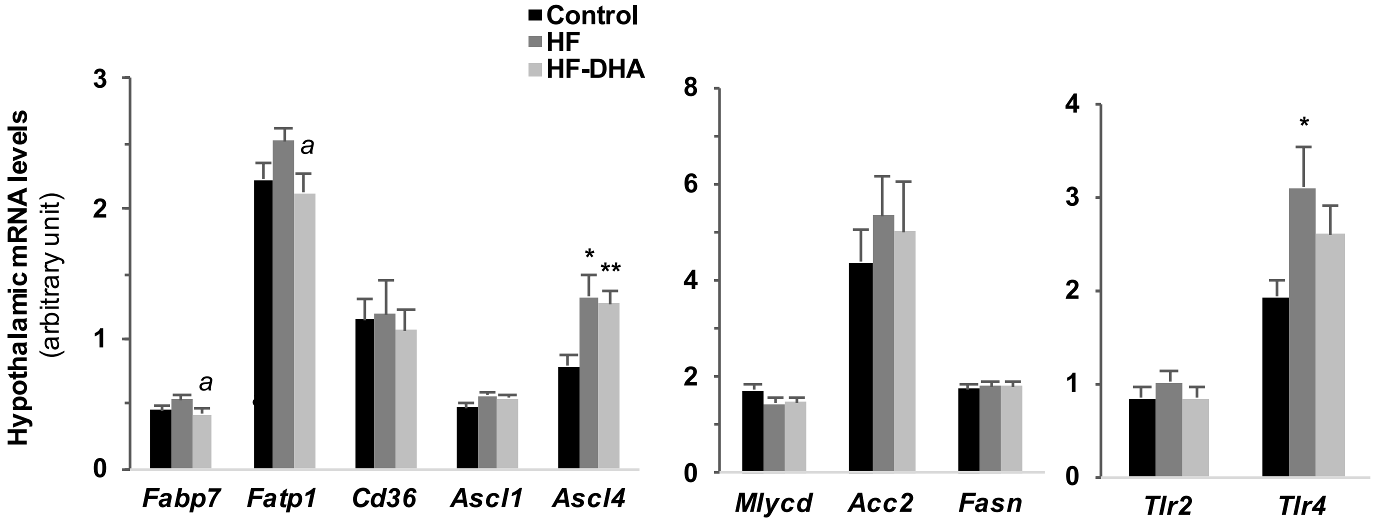

Supplement: Supplementary Figure 4 — Expression levels of genes coding proteins involved in the transport, metabolism or action of FA in anterior pituitaries and hypothalamus of rats fed short-term high-fat diets. The transcript levels of several genes were determined in pituitary (A) and hypothalamus (B) of rats fed short-term HF diets: genes involved in cell transport of FA: Fabp7, Fatty acid binding protein 7; Fatp1, Fatty acid transport protein 1; Cd36, fatty acid transporter/cluster of differentiation 36; Acsl1, Acyl-Co1 synthetase long-chain family member 1; Acsl4, Acyl-Co1 synthetase long-chain family member 4; genes involved in FA metabolism: Mlycd, Malonyl-CoA decarboxylase; Acc2, Acetyl-CoA carboxylase 2; Fasn, Fatty acid synthase and genes mediating FA inflammatory effects: Tlr2 and Tlr4, Troll-like receptor 2 and 4. Data are expressed as means ± SEM (n= 11-12 rats) and were analyzed with one-way ANOVA followed by Tukey’s multiple comparison test. *P ≤ 0.05; **P ≤ 0.01compared with control group.; aP ≤ 0.05 between HF and HF-DHA groups. [file DataSheet_4.pdf]
