# Supplementary material for: Disruption of Pituitary Gonadotrope Activity in Male Rats After Short- or Long-Term High-Fat Diets Is Not Associated With Pituitary Inflammation
Source: Front Endocrinol (Lausanne). 2022 Apr 13;13:877999. doi: 10.3389/fendo.2022.877999 (PMC9043610; doi:10.3389/fendo.2022.877999)
Supplement: Supplementary file 6 [file DataSheet_6.pdf]

Supplemental table 1: Composition of high fat diets (246HF SAFE)

g/kg	246HF	246HF+5% DHA
Maltodextrin	219.38	173.53
casein	228	213
Saccharose	206.3	206.3
Algae DHA Gold	0	120
Butter (anhydrous milk fat)	225	190
minerals PM AIN 93M/G 3,5%	45.5	45.5
Soybean oil	25	25
Vitamins PV AIN 93M/G 1%	13	13
Sodium bicarbonate	10.5	5.65
Potassium citrate	5,5	4
Choline bitartrate	2	2
DL-méthionine	2	2
Butylhydroquinone	0.02	0.02
Calcium carbonate	17.8	0
Protein %	19.8	19.8
Fat %	25.2	25.1
Minerals %	6	6.3
Cellulose %	0	0.7
Starch %	18.7	18
Sugars %	24.6	24.2
ENA %	46.2	44.7
ATWATER Kcal/Kg	4902	4842.7
ATWATER MJ/kg	20.5	20.3
E (energy)		
Protein Kcal/kg	791.9	791.4
Fat Kcal/kg	2263.8	2263
ENA Kcal/kg	1846.3	1788.3
E from protein %	16.2	16.3
E from fat %	46.2	46.7
E from carbohydrates %	37.7	36.9
Fatty acids profile mg/kg		
Saturated FA	158481	145329
Unsaturated FA	69783	76116
Mono-unsaturated FA	49555	43119
C18:1 ω9, (FFA/LFA) oleic acid and isomers (9Z-octadécénoic)	44612	38871
Poly-unsaturated FA	20228	32997
C18:2 (AL) ω6 linoleic acid	15700	15845
C18:3 (ALA) ω3 α-linolenic acid	2663	2605
C20:3 (DGLA) ω6 dihomo-gamma-linolenic	142	120
C20:4 (AA) ω6 arachidonic acid	162	137
C20:5 (EPA) ω3 eicosapentaenoic acid	61	423
C22:6 (DHA) ω3 docosahexaenoic acid	0	12600
Total Omega 3	2823	15712
Total Omega 6	16004	16102
% DHA relative to lipids	0	5
ω6/ω3	5.7	1
mg/kg		
Na Sodium	4258	4207
K Potassium	6950	6911
Ca Calcium	13360	13304
P Phosphorus	3360	3501
Mg Magnesium	1009	944
Mn Manganese	15	15
Fe Iron	64	64
Cl Chloride	1904	2008
